# Grass Gazers: Using citizen science as a tool to facilitate practical and online science learning for secondary school students during the COVID‐19 lockdown

**DOI:** 10.1002/ece3.6948

**Published:** 2020-11-24

**Authors:** Shanice Van Haeften, Andelija Milic, Beth Addison‐Smith, Christopher Butcher, Janet Mary Davies

**Affiliations:** ^1^ School of Biomedical Science Centre Immunity and Infection Control and Centre for the Environment School of Biomedical Science Queensland University of Technology Brisbane Qld Australia; ^2^ Agricultural Farm and Science Innovation Centre Corinda State High School Brisbane Qld Australia; ^3^ Office of Research Metro North Hospital and Health Service Brisbane Qld Australia

**Keywords:** citizen science, collaboration, COVID‐19, engagement, grass identification, pollen monitoring

## Abstract

The coronavirus disease of 2019 (COVID‐19) pandemic has impacted educational systems worldwide during 2020, including primary and secondary schooling. To enable students of a local secondary school in Brisbane, Queensland, to continue with their practical agricultural science learning and facilitate online learning, a “Grass Gazers” citizen science scoping project was designed and rapidly implemented as a collaboration between the school and a multidisciplinary university research group focused on pollen allergy. Here, we reflect on the process of developing and implementing this project from the perspective of the school and the university. A learning package including modules on pollen identification, tracking grass species, measuring field greenness, using a citizen science data entry platform, forensic palynology, as well as video guides, risk assessment and feedback forms were generated. Junior agriculture science students participated in the learning via online lessons and independent data collection in their own local neighborhood and/or school grounds situated within urban environments. The university research group and school coordinator, operating in their own distributed work environments, had to develop, source, adopt, and/or adapt material rapidly to meet the unique requirements of the project. The experience allowed two‐way knowledge exchange between the secondary and tertiary education sectors. Participating students were introduced to real‐world research and were able to engage in outdoor learning during a time when online, indoor, desk‐based learning dominated their studies. The unique context of restrictions imposed by the social isolation policies, as well as government Public Health and Department of Education directives, allowed the team to respond by adapting teaching and research activity to develop and trial learning modules and citizen science tools. The project provided a focus to motivate and connect teachers, academic staff, and school students during a difficult circumstance. Extension of this citizen project for the purposes of research and secondary school learning has the potential to offer ongoing benefits for grassland ecology data acquisition and student exposure to real‐world science.

## Graphical abstract

The coronavirus disease of 2019 (COVID‐19) pandemic has impacted educational systems worldwide, in particular primary and secondary schooling. To enable students of the local secondary school in Brisbane, Queensland, to continue with their practical agricultural science learning and facilitate online learning, a small‐scale citizen science project was designed and rapidly implemented as a collaboration between the school and a multidisciplinary university research group focused on pollen allergy. This study reflects on the process of developing and implementing this project from the perspective of the school and the university, the challenges and benefits incurred by both parties as well as the potential future implementations of this project for school learning and grassland ecology data acquisition.

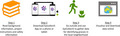

## 1 INTRODUCTION

As a response to the COVID‐19 pandemic, declared by the World Health Organization on 11th of March 2020 (WHO, 2020), several public health strategies have been adopted and applied in Australia, including restriction on international air travel, case isolation and home quarantine, social distancing, and school closures (Chang et al., 2020). Most schools across the country temporarily closed, while other schools were only open for children of essential workers. State governments encouraged parents to keep their children at home (NCIRS, 2020). This resulted in educators from primary to tertiary sectors needing to transition to online learning. This transition to online learning significantly hindered delivery of science curriculum that depends on practical activities to facilitate learning.

Announcement of the COVID‐19 pandemic lockdown in Queensland, Australia, caught Queensland University of Technology's Allergy Research Group (QUT ARG) and Corinda State High School (CSHS) in the early stage of consideration of a joint scientific field activity, which would act as a scoping study for a much larger citizen science (CS) project. While initially preparing to postpone the activity until after the social lockdown measures had ceased, the idea was formed to restructure it so that students could participate in a joint activity from home during lockdown and in a way that kept students engaged and able to undertake practical science learning. This led to the development of a small‐scale “Grass Gazers” CS scoping project and educational modules using “real‐world” research concepts and data to facilitate practical and online learning.^1^ Citizen science refers to a process whereby volunteers from within the community are able to engage in scientific research through assisting in the collection of data and sharing information. These types of projects not only enable the community to engage in research, but also allow researchers to achieve their broader research goals (Jordan et al., 2011; Silvertown, 2009). With the recent advancements in digital technology, there is now the potential for citizen science projects to recruit a larger number of volunteers across the world (Kobori et al., 2016) and there are an increasing number of freely available online tools for collecting and visualizing data (Dickinson et al., 2010; Silvertown, 2009). Access to these facilities coupled with the immediate need for online learning opened an opportunity for engaging secondary school students in interactive science learning activities while they were undertaking online school learning at home.

The scoping project was designed and developed by the QUT ARG for the purpose of expanding knowledge on distribution of local grasses in flower (Davies et al., 2015; Devadas et al., 2018). The project was adapted by the CSHS agricultural science coordinator as a learning tool suitable for use by a group of junior high school agricultural science students. The adaptation allowed for examination of the potential for use of this citizen science tool to engage with students working from home while facilitating practical and online learning. It also provided an opportunity to develop an ongoing collaboration between tertiary researchers and secondary educators. The project used online tools to upload botanical and phenological data of grasses observed by students around their neighborhood or school surrounds within an urban environment. The students could then access and visualize all collected data for further educational purposes. This paper will reflect on the development, implementation, current social context, and outcomes of this project for the school students, including the collaboration's challenges and benefits, how the project could be improved, and implications for future science learning in schools.

## 2 MOTIVATION FOR THE PROJECT

Information regarding grass species distribution records is documented by herbarium sightings collated by the Atlas of Living Australia (Atlas of Living Australia, 2020a), but this does not include phenological data on flowering behavior at a local level in Australia, which is still not well documented. Addressing this knowledge gap is valuable because grass has been recognized as a clinically important outdoor pollen producer globally (García‐Mozo, 2017) and the most common allergenic pollen source in Australia (Davies, 2014). Grass pollen has been shown to be a major trigger for Allergic rhinitis (AR), a disease that impacts over 500 million people worldwide, and as many as 19.3% of Australians (AIHW, 2019; Bousquet et al., 2008). Symptoms of AR such as itchy eyes and a runny nose can affect the health and quality of everyday life (Rimmer & Davies 2015) as well as impose a considerable socio‐economic impact (Colás et al., 2017). AR is also a known risk factor for progression to comorbid conditions such as sinusitis, sleep apnea, and asthma (Bousquet et al., 2019; Davies, 2014; Guerra et al., 2002).

One method to assist AR sufferers and clinicians, with public environmental health strategies to manage allergen exposure and symptom reduction, is the establishment of pollen monitoring networks to provide local current pollen information (Medek et al., 2019). Currently, there are 14 out of a total of 25 pollen monitoring sites of the AusPollen Aerobiology Collaboration Network (Davies et al. 2016) across Australia that publicly report daily grass pollen concentrations during the pollen season (NEII, 2020). Airborne pollen and spores are monitored by trained personnel by light microscopy by standardized protocols (Beggs et al., 2018). The process of pollen monitoring is labor‐intensive, costly, and time‐consuming, and moreover, the reported concentrations disseminated to community reflect yesterday's rather than today's conditions (Banchi et al., 2019; Thibaudon et al., 2013). A further problem with reporting airborne pollen concentrations for a large area such as Brisbane is that spatial variation is predicted to be high, but the representation of pollen monitored at one or a few sites over a wider geographic area remains poorly understood (Katelaris et al., 2004). The Brisbane area has a subtropical climate but incorporates a range of diverse microclimates based on urban development including bayside, riverside, urban, peri‐urban, and semirural areas. Historically in Australia or elsewhere worldwide, the subtropical grass pollen season (Green et al., 2004) has not been as well studied as the temperate regions. An improved understanding of local grass diversity, distribution, and flowering behavior around Brisbane would augment pollen forecast and alert systems. Addressing these paucity of data is important for aerobiology research purposes, but needs a novel approach because of the geographic scope. This motivated QUT ARG to consider a CS method. The first step toward data collection would be a scoping study aimed to evaluate tools, methods, and training materials, rather than to generate a broadly useful dataset.

**FIGURE 1:**
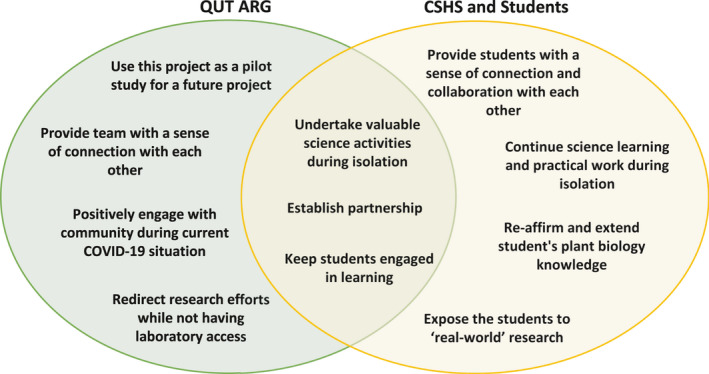
Summary of individual and collective drivers of motivation for engaging in the project from the perspective of each partner organization

The external context of the COVID‐19 situation required the CSHS to abandon activities such as student camps and many of the usual practical facets of agricultural studies. Teachers were required to rapidly adopt online learning formats and create material for the delivery of their secondary science curriculum. These conditions created an opportunity for CSHS and QUT ARG to rapidly develop a proxy for the proposed scoping study, allowing researchers to test methods and material and creating valuable content and experiences for the students. While there were both internal and external drivers that motivated the QUT ARG and CSHS to proceed with this project, there were also shared purposes that underpinned this collaboration (Figure 1). Within the current circumstances, both QUT ARG and CSHS recognized an opportunity to develop and implement an online CS project and provide a platform to keep students engaged during the COVID‐19 isolation period. Moreover, the use of technology underscores the potential to increase the engagement and motivation of learners and allows catering to different learning styles (Jones & McLean, 2012).

For CSHS, this project provided the opportunity for their students to remain engaged in learning and feel a sense of connection with each other while at home during lockdown. Even though they did not share the same physical space, they still functioned as a class via use of online learning platforms. All the students were potentially able to add data to a shared online database and view each other's entries online. The activities allowed students to reaffirm and extend knowledge of plant biology topics previously taught. The students were exposed to “real‐world” scientific research, authenticating learning and encouraging development of reflective and critical thinking skills.

The COVID‐19 restrictions resulted in the QUT ARG members having to work from home, requiring them to redirect their research efforts while they were unable to undertake laboratory work. Engaging in this project also required the researchers to regularly connect and collaborate via online tools. This project provided opportunity to test and refine tools for a proposed CS research project on grass distribution including testbeds for data collection methods and analysis. The data collected from this project could serve the group's research goals, including the generation of better grass pollen forecasts. Undertaking and reflecting on the project allowed the research group to improve the research and engagement tools prior to larger scale application.

The aim of the collaborative “Grass Gazers” project was to provide learning materials and an online tool for students to undertake practical science activities safely around their homes. Moreover, this project would enable CSHS and QUT ARG to establish a new collaboration involving elements in both secondary and tertiary education sectors.

## 3 PROJECT DESIGN

**FIGURE 2:**
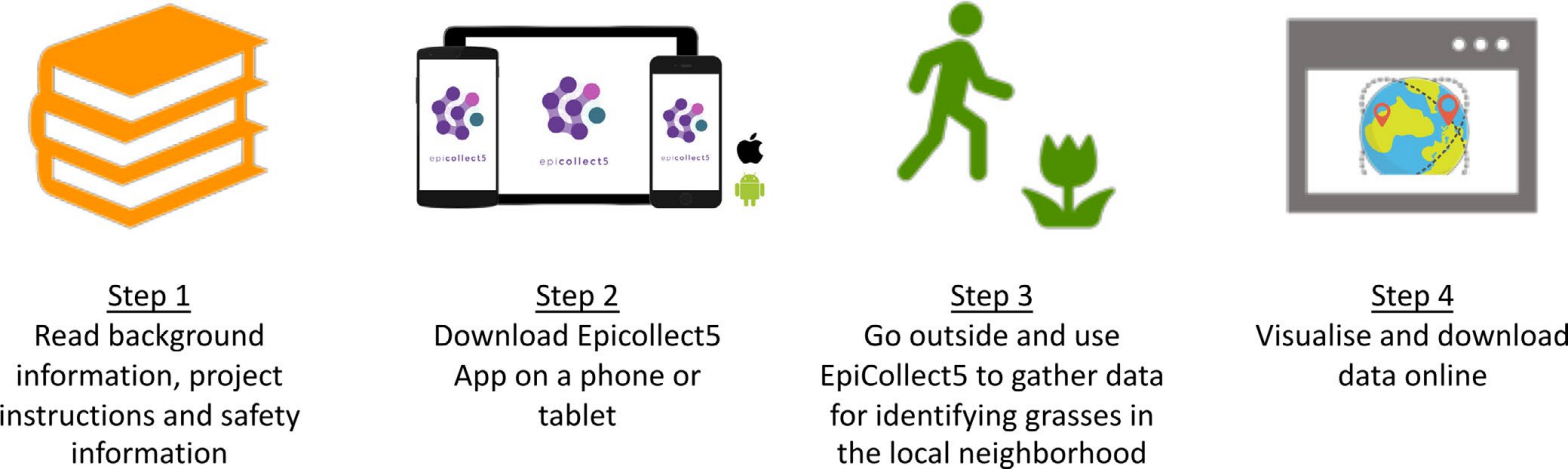
Overview of the practical "Tracking Grass Species" activity

The collaborative “Grass Gazers” CS project was designed to geolocate, characterize, and indicate flowering behavior of grasses around students’ homes through an activity named “Tracking Grass Species” (Figure 2). Before the students began undertaking the practical activity, it was important that they had background knowledge on the project and safety information as well as instructions on how to undertake the data collection. The QUT ARG provided the agricultural science coordinator with a set of resources (documented versions of the developed risk assessments and the modules on pollen identification, tracking grass species, measuring field greenness, using a CS data entry platform, and forensic palynology) covering this information. The science coordinator then used this information to generate online class lessons that incorporated a range of teaching tools (e.g., questions, activities, images using simplified language) to help the students understand the project and the risks involved. These tools included developing the learning package with instructional videos to show students how to download the data collection app and then upload data onto the App, adding multi‐choice and open ended questions as well as table questions to compare similarities and differences. This was done using an online platform called “Stile” (Stile Education, 2019) with functionality that included uploading videos, multi‐choice and open questions, mind maps, an open canvas function to annotate images and audio commentary. Generating online lessons in this format allowed students to interact with the theory. Other topics discussed in these online lessons included examining hazards and putting them in context in which the students were collecting specimens and looking at the morphological features of different pollens. Based on the project risk assessments, the students’ parents were also informed of the activity risks and mitigation strategies associated with undertaking the practical activity. Once the students were familiar theory and with important safety information, they were then able to begin the practical component of this project. The project required the students to look outside their household or around their local neighborhood, under supervision, and locate and characterize flowering grass species using an online data collection platform, Epicollect5. This CS platform is free to use on mobile and web applications and is run and managed by the Oxford Big Data Institute. This platform allows project administrators to generate forms (surveys) to collect an unlimited volume of data, including GPS and media, from users. The entire dataset can be viewed online (either publicly or in hidden mode) via maps or tables, and the data can be exported in different formats (EpiCollect^2020^, ^2020^).

The QUT ARG developed a project on this platform with an online survey that asked about the grass species observed (Appendix S1). Supporting information included key phenological characteristics and images that provide a significant amount of detail to enable students and researchers to identify the type of grass flower head, leaf shape, and leaf arrangement that the students were observing. In further questions students could attempt to identify their grass and were provided with images of common local grasses. Further questions involved broadly describing the grass site and estimating the grass diversity of the site. As the students submitted data and photo images into Epicollect5, they were able to visualize data collected by themselves and their school classmates. This online platform also allows the student to visualize the geographic location of all data on a map. The data collected can be exported by the students for further educational activities developed by their science teachers.

## 4 PROJECT OUTPUTS AND OUTCOMES

There were a number of key outputs generated. The first output was the development of a data collection survey that could be easily accessed via an online platform. Once the survey was set up onto EpiCollect5, it was ready for active use and the results can be easily visualized and analyzed. This survey can be adapted and expanded for more users.

Another output was the development of a suite of learning resources and activities for junior high school students between the ages of 13 and 14 years old. These resources can be used and adapted for future students at this and other schools. With slight modifications, these resources can be adapted appropriately to suit students of different ages (e.g., senior high schoolers and university students).

Lastly, a new small CS dataset was generated by students at CSHS who used these activities. During this trial, 39 data points were entered into the EpiCollect5 tool and these points were distributed across Brisbane (within a ~100 km area). Out of these 39 data points, 23 entries entered were correctly of grass, 3 were not grass, and 13 entries were missing the images of the grass thereby hindering the quality of the entry.

These outputs led to students being able to participate in a real‐world science activity with relevance to their own studies. These students learnt the features and structures used to identify plants (pasture grasses) and applied this knowledge to describe the plants they had found. This formed an important part of the plant component of the agricultural science course. Students participated in field work using mobile devices and in the creation of a shared dataset.

## 5 OUR EXPERIENCE—REFLECTION ON PROJECT DEVELOPMENT AND IMPLEMENTATION

The engagement between QUT ARG and CSHS was initiated prior to COVID‐19, and the intention to plan this CS project had already been considered. Subject to receipt of external funding, the project was to take a year to execute, with time devoted to plan and implement the project. Due to current social factors, the COVID‐19 situation and isolation policies, there was a desire for practical science activities for students to undertake while learning from home. This prompted rapid adaptation of the initial project concept to suit the needs of the school and accelerate the trial of proposed QUT ARG CS research tools.

**FIGURE 3:**
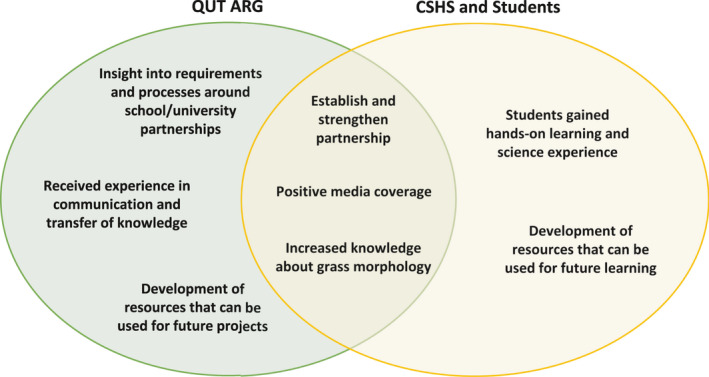
Summary of individual and collective benefits obtained from undertaking partnership/project

Throughout project development and implementation, several challenges were encountered, some of which were solely due to the COVID‐19 situation (Table 1). However, by engaging in this new collaborative project, the organizations gained both expected and unexpected benefits (Figure 3). These challenges and benefits will be discussed in the following sections.

**TABLE 1:** Summary of challenges that arose during project design and implementation and the mitigation strategies to overcome them

Challenges	Mitigation strategies
Limited time and resources	Multidisciplinary team to share ideas and support Members of team worked on the project in‐kind outside of work
Students not having access to resources (e.g., electronic devices and Internet)	Providing paper resources and worksheets
Ability to engage students and encourage participation	Email parents to let them know that their child needed assistance
Providing learning support for students	Development of detailed visual media
Health and safety concerns	Preparation of risk assessment document Email to parents/guardians advising them of risks
Governing body regulations and guidelines	Restriction of project scope to learning activities

### 5.1 Challenges

#### 5.1.1 Limited time and resources to develop and implement project

The QUT ARG had been preparing funding applications for the project at the time of lockdown. During the initial time frame, the QUT ARG planned to consider all aspects of the project prior to commencement of activities. However, due to the cessation of face‐to‐face schooling and closure of research laboratories, a majority of the CSHS school students quickly moved to learning from home, impacting the delivery of the agricultural science curriculum. There was a limited time and resources to develop new educational resources and tools needed to enable the students to undertake this project at home. Having a multidisciplinary team quickly facilitated development of the necessary educational resources and CS tools.

Without direct funding, members of the group worked on this project with considerable discretionary effort, and in their own time. Universities were undergoing their own transition to working from home, and this added administrative workload and logistical challenges. Having a new and engaging project, which had potential to directly helped the school students, gave a sense of altruistic purpose that connected the team during this disruptive period. Moreover, utilizing an existing data collection platform, like EpiCollect5, enabled the QUT ARG to save resources (i.e., finances and time) associated with developing a completely new platform for the project.

#### 5.1.2 Student access to resources was variable

Students having limited or no access to electronic resources (i.e., electronic device and Internet) were another major challenge. The original plan was to make use of a class set of iPads and to use the school‐based Internet for uploading data and photographs, and for viewing results. For loading data anonymously (essential for an activity involving school students), the Epicollect5 system uses a free mobile App; this means that at home, the students needed access to a mobile device of some kind, preferably with location turned on (for mapping) and with access to Wi‐Fi or data for uploading their observations.

Between 2016 and 2017, 91% of Australian households with children under 15 years of age had access to the Internet and 91% of these connected households used computers or mobile phones, while 66% of households also connected to the Internet using tablets (ABS, 2018). These statistics indicated that a small number of students may not have Internet access. This would impact the overall effectiveness of deploying this type of CS project as it relied on entering data into an online tool. The way the project was delivered to the students by the school was via an online learning platform.

A version of the learning resources that could be printed out was also provided to the students. These resources included background information and instructions on how to conduct the project as well as data entry sheets that would enable the students to collect data and then submit the information to their teacher or online when they eventually had access to an electronic device.

#### 5.1.3 Ability to engage students and encourage participation

Having the students learn from home for an extended period of time without constant contact with the teacher posed a challenge of effectively engaging the students and urging them to participate in the project. It was difficult for the agricultural science coordinator to monitor whether the students were completing the required schoolwork from home because they were not able to communicate with the students regularly. It was imperative that the students were self‐motivated to undertake the project and/or had someone available in their home to assist or enforce them to undertake the project. However, if the students lacked that motivation or someone in their home did not have the skills or time to assist the student with their work, then this could result in a reduced number of students participating.

To resolve this problem, the agricultural science coordinator contacted the student's parents/guardians via email if these activities had not been “opened” online to assist and encourage the student to complete the project activities. It was expected that the students would complete the activity as part of their online lessons but because there was no way of enforcing participation, the activity was not assessed.

#### 5.1.4 Providing learning support to students

This project required the installation and use of an online tool, EpiCollect5, which the school had not used before. Teaching the students how to download and use this tool was a further task that many students could find daunting, even in a supportive classroom environment. Moreover, there were students in the class who would struggle with learning via extended written instructions.

Online visual media (i.e., instructional videos) were used to assist the students in understanding how to install and use EpiCollect5. These videos provided step by step visual instructions, that the students could pause, and rewatch as often as they needed. Unfortunately, students who lacked access to an electronic device or the Internet needed to rely on the physical resource documents and the support of their guardian.

#### 5.1.5 Health and safety concerns

A risk assessment appropriate for the project was needed that encompassed both risk mitigation approaches taken by QUT and CSHS, and to determine appropriate level of responsibility. There are polices and legislative responsibility for ensuring health and safety of staff, adults, and students and processes for risk management of the projects led within Universities. Risk assessments established by university researchers are comprehensive, and based on adult engagement in activities, decision‐making and careful realistic assessment of the project‐related risks with an aim that all individuals involved in the project are aware of risks and mitigation strategies, and share in the responsibly to maintain safe work practices. A risk assessment document had to ensure that all project‐related risks are considered, and all risks are evaluated and managed appropriately for junior high school aged students. The schools, however, have different approaches to ensure health and safety of students, as normally students would carry out the project activities at school in an environment where supervision is provided, risk reduction strategies can be implemented and monitored.

Online and practical learning at students' homes and/or local environment brought a new health and safety challenge because the teacher could not monitor or enforce any of the measures outlined in the risk assessment document. The students were minors needing a degree of parental supervision. The QUT ARG prepared a risk assessment document with suggested considerations and guidelines regarding identified safety and risk mitigation plans for the project, as seen from the perspective of researchers to share with CSHS (Table 2). The version of the document was then considered, modified, and adopted into a simplified but effective form by CSHS. The school took the responsibility to email the students' parents/guardians to make them aware of the project and highlight all of the risks identified. The core recommendation was to always have students accompanied and supervised by a responsible adult. In addition, the main risks and health and safety measures were incorporated in online lessons and explained by the agricultural science coordinator before students had undertaken the activity. The EpiCollect5 online tool itself contained a short note on safety in the introduction.

**TABLE 2:** Identified hazard and risks associated with this project and the control measures recommended to overcome them

Hazard	Risk	Control
Working in isolation	Potential slip, trip, fall injury when no one may be able to assist. Negative effect of pre‐existing medical conditions of students	Provide initial safety information prior to activity Be accompanied by an adult or advise them of your whereabouts Be familiar with surroundings
Working in sun and heat	Heat exhaustion Dehydration Sunburn	Be accompanied by an adult or advise them of your whereabouts Limit amount of time to conduct identification activity Wear sun protection Carry water and mobile phone
Working in bad weather	Get caught in rain and/or thunderstorm	Be accompanied by an adult or advise them of your whereabouts Be familiar with forecasted weather Be familiar with potential shelters Postpone activities if weather is too severe
Potential car accident	Potential car accident injuries	Be accompanied by an adult or advise them of your whereabouts Carefully access the area and follow road rules Stop, look and listen for traffic Select alternative route
Potential snake, spider or insect bites	Could be bitten by snakes, spiders or other insects	Be accompanied by an adult or advise them of your whereabouts Be aware of exposure and presence of bite hazards Inspect area before performing activity Look before putting hands into vegetation
Current COVID‐19 situation	Be exposed to or contract virus	Students will comply with recommendations by the Queensland Government Students will practice social distancing
Online cyber safety	Cyber stalking risks with students entering information about plants in their area	Identity of students will be anonymous and nonidentifiable

#### 5.1.6 Considering governing body requirements

The QUT ARG needed to evaluate the project and partnership to determine whether this project complied with guidelines and policy and consider whether ethics approval would be required (NHMRC, 2018). During the COVID‐19 situation, the Queensland state government Department of Education issued a directive that all research activities in state schools and other educational sites were to be postponed until further notice (Government Queensland, 2020). Instead of research, the project aim was to develop learning activities for school students to conduct and raise awareness of research processes. Careful consideration throughout the development and implementation phases of the project was taken to comply with government regulations including frequently changing public health directives. It may be possible in future when the Department of Education directive changes, and if ethics approval is sought, to gather survey feedback from the students themselves on their perception of the learning opportunities and material provided.

### 5.2 Benefits

Given that the collaboration between QUT ARG and CSHS was established not long before the COVID‐19 situation arose, communication between parties was incredibly smooth including connecting by videoconferencing to develop engagement plans, learning materials, and an online platform. This project provided an opportunity to establish and strengthen new relationships between individuals working within these organizations that may underpin further activities and projects.

Throughout the development of this project, one of the anticipated benefits was the opportunity for Agricultural Technology students to enhance their knowledge about plant morphology and the identification of plants (grasses) in a real‐world context. However, unexpectedly, through the process of developing learning modules for the students, individuals from different disciplines within the QUT ARG (mathematics, environmental chemistry) consolidated their own knowledge about plant morphology and identification.

The QUT ARG team actively learnt about topics relevant to new aspects of the CS project that were applied and tested. The QUT ARG team gained valuable knowledge and insight on the requirements and processes for school–university partnerships. The project had the added benefit of providing early career researchers a lived experience of communication and transfer of knowledge to junior high school level.

The set of resources developed and refined here in will be used as a valuable teaching and learning material for future projects. These learning resources will allow for expansion of this project to extend CS involving other schools and community groups. Working with the school community has increased awareness of CS not only with students but also with their friends and families. This can be an important forum for a broader public engagement that will allow growth of awareness around the importance of grass diversity, phenology, grass pollen, as an allergy trigger, and management of pollen allergies.

With this CS project, students were given an opportunity for a hands‐on experience in grassland ecology field work and online data entry to a digital platform while learning at home. The designed online learning and practical activities have exposed students to real‐world science and likely facilitated a development of important scientific skills in observation, critical thinking and analysis. Students had to carefully observe features of grass, its flowers and leaves, decide on type of flower, flower head, suggest the likely grass species, and analyze generated data (Appendix S1). Learning approaches such as this are proven to be effective in increasing student interest in science, technology, engineering, and mathematics careers (Hiller & Kitsantas, 2015). CS projects, like this one, can be considered as an important initial step toward professional development of young individuals.

A further collective benefit was media coverage including a national television nightly news channel as well as university and school media platforms (QUT ARG, 2020). This coverage provided positive attention to all partners involved and increased awareness of science to the community including the project motivation and scope, as well as some of the health impacts of grass pollen.

Although time to develop and implement the project was limited, this facilitated the rapid evolution of the project because all individuals involved had a heightened focused on making things happen. This benefited all parties; the school teaching staff, university researchers and school students, and above all, inspired the team to provide a unique and much needed learning experience for students in difficult times.

## 6 DISCUSSION AND FUTURE DIRECTIONS

Citizen science has proven an effective method of leveraging cheap technology and community goodwill to gather data that would otherwise be impossible or prohibitively costly to collect (Bonney et al., 2016; Jordan et al., 2011; Silvertown, 2009). It dovetails into the concept of place‐based education which has benefits in improved learning and community identity (Miles, 2008; Sobel, 2004). CS is seen as a useful educational tool both for students and adults (Edelson et al., 2018; Kim et al., 2016), and one which supports proenvironmental changes in both students and the community (Haywood et al., 2016; Wals et al., 2014). To maximize success, a CS project still needs resources and careful design (Bonney et al., 2016; Edelson et al., 2018; Herodotou et al., 2018; Naqshbandi et al., 2020), ideally including preliminary trials. CS projects involving school students may require greater investment in quality control (Castagneyrol et al., 2020; Saunders et al., 2018).

To our knowledge, no CS projects have been published looking specifically at distribution and flowering of grasses, although some CS projects include observations of grasses as part of a broader scope (e.g., invasive weeds, Queensland Government, 2020). There is no national scale phenological network or systematic phenological data collection scheme in Australia, although the Atlas of Living Australia collects CS plant and animal sightings via iNaturalist, and some of these sightings may have had phenology tags added (Atlas of Living Australia, 2020b). National and continent‐wide phenology networks exist in Europe, the United States, and the United Kingdom encouraging varying degrees of CS input in order to specifically document and track plant species phenology. In the United States, a well‐resourced national scale CS plant and animal phenology scheme, Nature's Notebook (USA National Phenology Network, 2020), is run by the USA National Phenology Network and the USGS. They register no large grass or grass pollen projects but one of their eight large campaigns focuses on a major source of allergenic pollen in Texas *Juniperus ashei*. In Europe, the Medical University of Vienna have coordinated CS allergy diaries with pollen (including grass pollen) modeling, pollen monitoring, and local weather observations to the extent where they produce individualized daily allergenic pollen forecasts via an App (Medical University of Vienna, 2020), but it does not include grass phenology. This does not extend to Asia, Australia, or the Americas.

The activity involving QUT ARG and CSHS was initially planned as a scoping study, but the trial was forced to rapidly change to suit COVID‐19 lockdown. The development of this project followed a build‐measure‐learn cycle process; a core component of the Lean Startup methodology, which is commonly used by entrepreneurs wanting to quickly create and manage a successful start‐up. The aim of this business methodology is to reduce waste by quickly understanding all of the processes and activities that are unnecessary (Ghezzi & Cavallo, 2020). In our case, everyone involved wanted to understand what activities, learning material, and tools were needed to get the users to understand and engage, without using a significant amount of resources. For example, QUT‐ARG was able to identify a free online data collection platform that had all of the functionalities needed and was simple to use, instead of wasting resources to develop a bespoke data collection platform. The build‐measure‐learn cycle involves building the most simplified version of the working product/service that is then tested with a small group of users. Following feedback, the cycle is repeated until a well‐developed product/service is generated. In the case of this project, QUT ARG and CSHS rapidly developed a set of basic activities and learning resources, intended to enable the QUT ARG to receive informative data on grass distribution and phenology, as well as be engaging and educational for the students. The students were able to undertake this project and provide feedback, through a classroom discussion. By undertaking this first cycle of build‐measure‐learn process, we were able to identify key areas of our project that need improvement and then modify different components based on this reflection. We are can now test this project again with the students, measure its success and learn. The value of undertaking this type of iterative process is that it will enable the quick develop a CS project that has a higher chance of being successful and sustainable, once it is scaled up within the community (Ries, 2011).

Although not all of the data from the current project could be used for future research, this CS project did provide a significant insight into what aspects need to be improved prior to further use. Overall, it was evident that the type of data collected could be highly useful in increasing knowledge of current local distribution of grasses in flower. The research team were able to identify grasses in flower from the data, but it is clear that species identification would need to be verified by experts. As more data are collected over time, the QUT ARG could view the distribution of the different grass species across Brisbane on an interactive map as well as grass phenology. The outcomes of this project suggest that further use and extension of this CS approach would therefore enhance our current knowledge of grass distribution and phenology, with benefits in tracking and forecasting allergenic pollen.

There were three major aspects of this project that were identified as needing further consideration and improvement from the perspective of the students: better‐targeted instructions, improved participant support, and a tangible use of the CS data.

First, clearer instructions on how to undertake the project are needed. It was evident that the students were able to follow instructions to complete survey questions; however, some of the required images were not uploaded, and sometimes, the wrong part of the grass was photographed, hindering the quality of the data. This is partly due to testing the survey tool on adults rather than children and highlighted the need for survey refinement against the target users. It was additionally clear that some students of this age can still find mobile Apps confusing. In this project, the agricultural coordinator at the school was aware that some students would struggle with extended written tests, he learnt during this project that twelve and thirteen year old's do not always have the ability to navigate their way through difficult tasks without the face‐to‐face assistance of a teacher. This highlighted the overall importance of teaching students how to deconstruct and address complex problems. It was also evident that lay language should be used to describe scientific concepts and transfer knowledge to public audiences, but writing in plain language is a skill that needs to be developed. The ability to write effectively in this way is necessary to increase the visibility, transparency, and impact of science in the community (Kuehue & Olden, 2015). From this perspective, the learning modules were written for an intended audience of junior high school students, but the language was further simplified by the secondary school teacher. The learning modules should now be transferrable for effective engagement of lay people in community for a wider CS project.

The second component is improving the support system for participants in this project. While the school and university team members used an online file sharing and messaging software to communicate, it would be advantageous to use an online communication and collaboration platform (e.g., Microsoft Teams, Slack, Discord) for the students to discuss, connect with peers, and upload files relating to this project. An online hub could provide a centralized location for all project resources and data. The use of this type of platform could provide students with peer to peer support to communicate any issues or questions. Additionally, it might be better to perform similar projects as group activities when this is possible.

The third improvement is consideration of how the students can extend use of the data after it is collected. Activities could be developed for the students to graph and analyze the collected data, gather other datasets online (e.g. local rainfall or temperature), and explore mathematical, physical, and biological relationships. While this scoping project involved students collecting data in their local area, it stopped short of tying the data to some meaningful endpoint; completing this step would have added purpose and improved student engagement and learning (Sobel, 2004).

In scaling to a larger CS activity, it is also important to consider resources needed as the collected dataset grows. Funding will be a required input to expand activity. For instance, identification of grass in flower is not difficult or time‐consuming, but verification of grass species is a labor‐intensive job for an expert. For basic evidence of grass presence and flowering, it may be possible to assign quality levels to data post hoc: For instance, data without photographs would have the lowest quality, data with unchecked photographs the next and fully verified data assigned the highest research quality level. A choice could then be made on which subset of the data to use for further research purposes or analyses.

From evaluating the effectiveness of working with a school to test this CS project, it was evident that working within this collaboration with a shared purpose was a mutually beneficial experience for both the academic research group and the secondary school science department, consistent with the key principle of engagement necessary for quality CS projects. All partners shared clear aims and expected outcomes that were defined at the start of the project and were realistic for the time and resources available. The highly collaborative nature of the partnership was an important part of its success. It was recognized and valued that everyone involved in the project contributed different expertise. To facilitate this collaborative relationship, “open communication” was maintained between everyone involved in the project, which ensured that there was a clear sense of shared ownership of the project (Mclaughlin & Black‐Hawkins, 2004). This school–university partnership is recognized to have all the important elements for success, and this project provided an opportunity to strengthen the new relationships which will likely evolve over time (Green et al., 2019).

Throughout development and implementation of this project, the QUT ARG and CSHS both learned about the process and regulations of engaging in a school/university partnership. It provided early stage researchers with insight on how they can effectively communicate their research themes to the general community, whereas CSHS gained insights on how to use vertical integration and university partnerships to help students learn Twenty First Century skills in a real‐world multidisciplinary research context.

A number of observations from this project may be useful to others in developing and running similar activities:

1. It is possible to rapidly develop a small CS project with existing tools and very limited resources, and to introduce it to young high school students without working face to face;
2. It is worthwhile to search for existing solutions to minimize the project development stage;
3. It is very important to test the survey instrument on the target demographic and to spend as much time as possible on clarity of instructions;
4. Data resulting from a CS project involving students will need at least a moderate amount of quality control;
5. Facilitating group work, even if virtual, would probably improve CS engagement and outcomes;
6. Developing tangible and relevant end points for the data use is valuable for engaging citizen scientists; Strengthened partnerships and other unexpected benefits can flow from projects which may not initially seem to be well aligned with the purpose of one or both teams.

As a project aiming to facilitate learning, it was considered a success because the students appeared to enjoy using the online tools during this project as well as learning about grass and pollen. The students were also able to learn about how this project related to the bigger “public health” context, which was a major motivator. However, it was observed throughout the data collection process that a proportion of the students did not engage with the activity. This was attributed to the students working without supervision at home.

The science coordinator at the school hopes to continue undertaking this project with the next cohort of junior high Agricultural Technology students in subsequent semesters. Overall, this project has been one of the highlights of the agricultural science course and this will only improve in the future.

While serving as a learning and engagement experience in the context of the unique social setting of the CoViD‐19 lockdown, undertaking this type of CS project opens the opportunity to re‐evaluate approaches to conventional science learning. Collaboration between secondary and tertiary education sectors deepens the nexus between teaching and community participation in real‐world scientific research. The “Grass Gazers” CS project can be modified and extended for use in other schools, community groups, and the general public.

## CONFLICT OF INTERESTS

The authors report no competing interests for this project. Outside the scope of this project, Professor Davies receives grant funding for related grass pollen allergy research from the Australian National Health and Medical Research Council (NHMRC AusPollen Partnership Project (GNT 1116107)) with matching cash and in‐kind cosponsorship from The Australasian Society for Clinical Immunology and Allergy, Asthma Australia, Bureau of Meteorology, Commonwealth Scientific and Industrial Research Organisation, Stallergenes Australia, Federal Office of Meteorology and Climatology MeteoSwiss, Switzerland, as well as the Australian Research Council, National Foundation for Medical Research Innovation with cosponsorship from Abionic Switzerland, The Emergency Medicine Foundation. In the past five years, Professor Davies has also received grant funding from QUT Catapult scheme, Queensland Health, Bureau of Meteorology and Victorian state Department of Health and Human Services. She is an inventor on patents assigned to QUT. Her institute has received Honorarium payments and travel expenses for education sessions and conference presentations from Stallergenes Australia, Wymedical, and Meda Pharmaceuticals.

## AUTHOR CONTRIBUTION

**Shanice Van Haeften:** Conceptualization (supporting); Project administration (equal); Writing‐original draft (lead); Writing‐review & editing (equal). **Andelija Milic:** Project administration (supporting); Writing‐original draft (supporting); Writing‐review & editing (equal). **Beth Addison‐Smith:** Conceptualization (equal); Methodology (lead); Project administration (equal); Writing‐original draft (supporting); Writing‐review & editing (equal). **Christopher Butcher:** Conceptualization (supporting); Project administration (supporting); Writing‐original draft (supporting); Writing‐review & editing (equal). **Janet Mary Davies:** Conceptualization (equal); Supervision (lead); Writing‐original draft (supporting); Writing‐review & editing (equal).

## Supporting information

Appendix S1

## Data Availability

As this is a paper that is reflecting on the development and implementation of a project and collaboration, there were no data used for statistical analysis. The data collected by the CSHS students can be accessed through https://five.epicollect.net/project/qut‐corinda‐grass‐mappers.
