# Supplementary material for: Grass Gazers: Using citizen science as a tool to facilitate practical and online science learning for secondary school students during the COVID‐19 lockdown
Source: Ecol Evol. 2020 Nov 24;11(8):3488–500. doi: 10.1002/ece3.6948 (PMC7753511; doi:10.1002/ece3.6948)
Supplement: Supplementary file 1 — Appendix S1 [file ECE3-11-3488-s001.docx]

**Appendix** Survey questions presented on the EpiCollect5 online tool for students to collect grass data.

| **Question** | **Type of Answer** | **Justification** |
| --- | --- | --- |
| Where is the plant (Latitude and Longitude)? | Short answer | Allows for the geolocation of the observed grass plant to be recorded and mapped. |
| What is the date? | Short answer | Provides record on when the data was obtained |
| Take a photo of the grass flower | Image | Provides a visual of a major physical characteristic that can help identify the observed grass |
| How would you classify the flower head? | Multiple choice | A major physical characteristic that can help identify the observed grass |
| How are the leaves arranged on the flower stalk? | Multiple choice | A physical characteristic that can help identify the observed grass |
| What shape are the grass leaves? | Multiple choice | A physical characteristic that can help identify the observed grass |
| Take a photo of the grass in its surroundings | Image | Provides a visual of whole grass plant and where it is located |
| Does your grass look like one of the common grass species in the provided document? If so which one? | Multiple choice | Allows students to identify observed grass based on common grasses normally found in Brisbane |
| Where is the grass growing? | Multiple choice | Provides insight into the habitat that this grass is growing |
| How much of this type of grass is in this area? | Multiple choice | Provides details on the abundance of that grass in that specific area |
| How many DIFFERENT types of grass are in this area? | Multiple choice | Provides details on the level of grass diversity in specific area |
| Please add any extra information that would be useful | Short answer | Allows students to add any other information that they think is useful or important |
